# Early detection of breast cancer in Brazil: data from the National Health Survey, 2013

**DOI:** 10.1590/S1518-8787.2017051000191

**Published:** 2017-06-01

**Authors:** Gulnar Azevedo e Silva, Paulo Roberto Borges de Souza-Júnior, Giseli Nogueira Damacena, Célia Landmann Szwarcwald

**Affiliations:** I Departamento de Epidemiologia. Instituto de Medicina Social. Universidade do Estado do Rio de Janeiro. Rio de Janeiro, RJ, Brasil; II Laboratório de Informações em Saúde. Instituto de Comunicação e Informação Científica e Tecnológica em Saúde. Fundação Oswaldo Cruz. Rio de Janeiro, RJ, Brasil

**Keywords:** Breast Neoplasms, diagnosis. Mammography, utilization, Early Detection of Cancer, Mass Screening, Health Surveys, Neoplasias da Mama, diagnóstico. Mamografia, utilização, Detecção Precoce de Câncer, Programas de Rastreamento, Inquéritos Epidemiológicos

## Abstract

**OBJECTIVE:**

To analyze whether the actions of early detection of breast cancer, initiated with the medical request for mammography, differ between users of the Brazilian Unified Health System (SUS) and those who have private health insurance.

**METHODS:**

From the data collected in the National Health Survey, we estimated the proportions of women who had medical request for mammography according to presence or absence of private health insurance. For assessing the factors related to having mammography medical request, we estimated crude and adjusted odds ratios and respective 95%CI by logistic regression. We also analyzed the main reasons reported for not having performed mammography after medical request, as well as the time between examination and result.

**RESULTS:**

Of the women interviewed, 66.7% had a medical request for mammography (59.4% among SUS users and 83.9% among those with private health insurance). Having private health insurance, higher education level, and being white were positively associated with having the medical request. Only 5.4% (95%CI 4.8–6.0) of women who received medical request failed to perform mammography – 7.6% were SUS users and 1.7% had health insurance. The most reported reasons for not being able to perform the examination were: not thinking it was necessary; having the test scheduled, but not yet performed; and not being able to schedule it. More than 70% of women received the result with less than one month from its execution.

**CONCLUSIONS:**

The barriers to access a medical request for mammographic screening for breast cancer are higher among women who depend exclusively on SUS.

## RESUMO

**OBJETIVO:**

Analisar se as ações de detecção precoce para o câncer de mama, iniciadas com o pedido médico de mamografia, diferem entre usuárias do Sistema Único de Saúde (SUS) e aquelas que possuem plano de saúde privado.

**MÉTODOS:**

A partir dos dados coletados na Pesquisa Nacional de Saúde, foram calculadas as proporções de mulheres que tiveram pedido médico para realização de mamografia segundo ter ou não plano de saúde privado. Para avaliar os fatores relacionados a ter pedido médico de mamografia, foram calculadas *odds ratios* brutas e ajustadas e os respectivos IC95% por regressão logística. Foram também analisados os principais motivos informados para não ter realizado mamografia após ter o pedido médico, assim como o tempo entre ter realizado o exame e o recebimento do resultado.

**RESULTADOS:**

Das mulheres entrevistadas, 66,7% tiveram um pedido médico para mamografia (59,4% entre as que eram usuárias do SUS e 83,9% entre as portadoras de plano de saúde privado). Ter plano de saúde privado, maior nível de escolaridade e ser de cor branca se associaram positivamente a ter o pedido médico. Apenas 5,4% (IC95% 4,8–6,0) das mulheres que receberam solicitação médica não conseguiram fazer mamografia – 7,6% eram dependentes do SUS enquanto 1,7% eram portadoras de plano de saúde. Os motivos mais reportados de não conseguir realizar o exame foram: não achar necessário; ter o exame marcado, mas ainda não realizado; e não ter conseguido marcar. Mais de 70% das mulheres receberam o resultado antes de completar um mês da realização.

**CONCLUSÕES:**

As barreiras de acesso a ter pedido médico para rastreamento mamográfico para o câncer de mama são maiores entre mulheres que dependem exclusivamente do SUS.

## INTRODUCTION

In Brazil, as in many other countries, breast cancer is the most common type of cancer among women. Mortality rates by the disease, estimated since 1980, show an increase in the five regions of the Country^1^. This increase can be explained, in part, by the increased incidence of the disease due to greater exposure of women to risk factors related to the urban lifestyle, which interferes directly in the gradient of exposure to reproductive factors. However, since the end of 1990, a decrease in mortality in the capitals of the South and Southeast regions can be observed. At the same time, a large annual increase in the rates of deaths among women from interior cities is perceived, mainly in the North and Northeast regions, which suggests that the reproductive and sexual changes, initiated in major urban centers, were quickly adopted by women of other cities, even in low-income areas. On the other hand, the reversal in mortality, which began in the capitals, became possible by the access to diagnostic and therapeutic means of women in the early stages of the disease^2^.

The results of large randomized clinical trials published in the 1970s and 1980s^3,4^ showed that mammographic screening would be able to reduce by 20%–30% the mortality by this malignant neoplasia. In fact, the introduction of population mammographic screening has contributed to decrease mortality in several developed countries, since the examination allows detection of tumors still in early stages of the disease, enabling the patients to have good response to the therapies available today^5,6^.

In 2004, the Brazilian Ministry of Health released the document of Consensus for Control of Breast Cancer, recommending the annual clinical breast examination as important action for detecting the disease to women from the age of 40 and the mammographic screening every two years to women between the ages of 50 and 69^7^.

Some surveys conducted in the Country since the 2000s estimated the coverage of mammography within the recommended age range from 50 to 69 years. The Household Survey on Risk Behavior and Reported Morbidity of Non-Communicable Diseases and Injuries, held in 2003 in 15 capitals, estimated that the mammography coverage in the two years preceding the survey varied between the surveyed cities – from 37% in Belém (PA) to 77% in Vitória (ES)^8^. In 2008, the National Household Sampling Survey (PNAD – *Pesquisa Nacional por Amostragem de Domicílio*) estimated a mammography coverage of 54.2% (95%CI 53.5–55.0) among women from 50 to 69 years in the two years preceding the interview, with a great difference between the North (35.3%) and Southeast (63.8%) regions^9^. In 2013, the National Health Survey (PNS - Pesquisa Nacional de Saúde) estimated, for this same age group, a 60.0% coverage (95%CI 58.8–61.3) for all Brazil, and a great variation amplitude between regions^a^.

Based on population surveys, one can verify that the coverage self-reported by women of the capitals of the Country, when compared to that of women living in other cities, was already greater since 2003. From PNAD 2003 and VIGITEL 2007 data, authors estimated that the coverage of the exam in Brazilian capitals was 70%, ranging from 41.2% in Porto Velho (RO) to 82.2% in Florianópolis (SC)^10^.

This scenario reinforces the hypothesis that the expansion of access to mammographic screening depends not only on the supply and quality of the exams, but also on the organization of the health-care network and accession of professionals to the existing guidelines. Thus, this study aimed to analyze whether the actions of early detection for breast cancer, initiated with the medical request for mammography, differ between users of the Brazilian Unified Health System (SUS) and those who have private health insurance.

## METHODS

For this study, we used data collected in the PNS carried out by the Brazilian Ministry of Health in partnership with IBGE, which aimed to assess the health, lifestyle, and health-care of the adult population in Brazil^11^.

PNS is a household survey that belongs to the Integrated System of Household Surveys (SIPD) of IBGE. It has a stratified and conglomerate sampling with three stages of selection. The census tracts formed the primary sampling units and, as part of SIPD, the selection of these units was obtained by simple random sampling, from the Master Sample. The stratification of the primary sampling units in PNS was the same used in the Master Sample^12^.

In the second stage, a fixed number of households was selected in each primary sampling unit, from the National File of Addresses for Statistical Purposes. In the third stage, within each household sampled, a resident with 18 years or older was selected at random from a list of eligible residents built at the time of the interview.

Of the 69,954 selected and occupied households in which residents were selected for interview, 60,202 individuals agreed to participate, which led to a response rate of 86%^11,13^.

PNS data were collected by trained interviewers using handheld computers (personal digital assistant). The selected adults responded to the questionnaire on sociodemographic characteristics, health self-assessment, lifestyle, morbidity, accidents and violence, women’s health, children’s (under two years) health, oral health, elderly health, and health system performance. In addition, anthropometric data blood pressure were measured and biological material was collected. More details on the sampling process and data collection of PNS were described by Souza-Junior et al.^13^.

The data of mammography for the population of women between 50 and 69 years were accessed in the module K of PNS “Health of individuals of 60 years or older and coverage of mammography among women of 50 years or older.”

The module R of PNS, regarding women’s health, contained questions on the following variables: “Having medical request for mammography” and “Having performed the examination after the request and receiving the result,” as well as the respective time between each situation and reasons for not having done the exam after medical request and the time between performing the exam and receiving the result.

Initially, we estimated the coverage of mammography among the target population of the screening (women between 50 and 69 years) from the proportion and respective 95% confidence intervals (95%CI) of women who reported having done the examination in the past two years, according to presence or absence of health insurance by regions of the Country. Then, we estimated the proportions and respective 95%CI of having medical request to perform mammography according to age group, with stratification between women using SUS and those with health insurance.

For assessing the factors related to having mammography medical request, we estimated crude and adjusted odds ratios (OR) and respective 95%CI by logistic regression. For this analysis, we selected the following factors: age group (40–49; 50–59; 60–69; 70 or older); education level (without education or some elementary school; elementary or middle school or some high school; high school or some higher education; higher education degree); region of the Country; race/skin color (white; nonwhite); and having private health insurance.

Among the women who could not perform mammography after having medical request, we estimated the frequencies and 95%CI of the most frequent reasons informed, according to presence or absence of health insurance.

Additionally, we estimated the distribution of women according to the difference in time between examination and result, separating SUS users and those with health insurance into the following categories: less than 1 month; 1**├** 3 months; 3**├** 6 months; 6 months or more; has not yet received; never received.

The analyses were carried out using the SPSS statistical program version 21.0^14^. For being a survey with stratification of the primary sampling units and selection by conglomerates in three stages, we considered the complex sampling design throughout the statistical analysis of data.

PNS was approved by the Human Subject Research Ethics Committee of the Brazilian Ministry of Health (Opinion 328,159, June 26, 2013). All participants signed the informed consent form.

## RESULTS

From the information reported in module K, among women from the target age group for screening breast cancer in Brazil (50 to 69 years), 60.0% (95%CI 58.8–61.3) performed a mammography in the past two years. This proportion ranged from 38.7% in the North region to 67.9% in the Southeast region. The proportion of women who performed the examination was much higher among those with health insurance (79.5%) when compared to SUS users (51.0%), condition that was repeated in all regions of the Country (Table 1).

**Table 1:** Coverage of mammography among women from 50 to 69 years with and without private health insurance, according to regions of Brazil. Brazil, 2013.

Region	All		Without health insurance		With health insurance	
		
	%	95%CI	%	95%CI	%	95%CI
North	38.7	36.0–41.5	31.9	29.1–34.8	69.5	63.5–74.9
Northeast	47.9	45.7–50.1	41.0	38.6–43.4	77.4	73.2–81.0
Southeast	67.9	65.9–69.8	59.3	56.8–61.8	81.4	78.8–83.7
South	64.5	61.4–67.5	56.6	53.1–60.1	80.0	74.4–84.7
Midwest	55.6	52.8–58.3	46.0	42.7–49.3	72.3	68.4–75.9
Brazil	60.0	58.8–61.3	51.0	49.5–52.4	79.5	77.6–81.2

Regarding the information on having medical request and referrals, contained in the module R, 66.7% of women have had a mammography request made by a doctor, and this proportion was higher (72.7%) for women aged between 50 and 59 years (Table 2). However, the percentage of women who reported having had medical request for mammography presented great difference according to presence or absence of health insurance (83.9% and 59.3%, respectively). In addition, a higher number of women among those with health insurance was performing mammography outside the age range indicated for the population screening. For women from 40 to 49 years, 84.5% with health insurance reported having received medical request, a significantly higher ratio than that reported for SUS users (58.1%). Similarly, for women with 70 years or older, 83.9% with health insurance had the medical request, while only 59.4% of SUS users had it. Among those with health insurance, we draw attention to the similar proportions of women between 40 and 49 years and 60 and 69 years (84.5% and 83.5%, respectively) with mammography medical request (Table 2).

**Table 2:** Proportion of women who reported having had medical request for mammography by age group, according to presence or absence of private health insurance. Brazil, 2013.

Age group (years)	All		Without health insurance		With health insurance	
		
	%	95%CI	%	95%CI	%	95%CI
40–49	65.9	63.9–67.7	58.1	55.8–60.3	84.5	81.4–87.0
50–59	72.7	70.3–74.9	66.2	63.3–68.9	89.5	86.2–92.1
60–69	68.2	65.5–70.8	61.3	58.1–64.3	83.5	78.6–87.4
≥ 70	54.9	51.6–58.1	45.7	41.7–49.6	74.1	68.8–78.6
Total	66.7	65.3–67.9	59.4	57.8–60.8	83.9	81.8–85.7

The reasons that were most positively associated with having had a mammography medical request were: having health insurance (OR = 2.60, 95%CI 2.20–3.07), being within the target age groups of the screening (OR 50–59 years = 1.47, 95%CI 1.27–1.71; OR 60–69 years = 1.20, 95%CI 1.03–1.41), and being white (OR = 1.23, 95%CI 1.08–1.40). Education level was also positively associated: the higher the education level, the greater the chance of having a mammography request. On the other hand, living in different regions than the Southeast presented lower chance of having mammography medical request (Table 3).

**Table 3:** Factors associated with having had mammography medical request. Brazil, 2013.

Factor	_crude_OR	95%CI	OR*	95%CI
Age group (years)				
40–49	1.00	-	1.00	-
50–59	1.38	1.20–1.59	1.47	1.27–1.71
60–69	1.11	0.96–1.29	1.20	1.03–1.41
≥ 70	0.63	0.54–0.74	0.66	0.56–0.79
Education level				
Illiterate/some elementary or middle school	1.00	-	1.00	-
Elementary or middle school/some high school	1.56	1.32–1.84	1.27	1.06–1.52
High school/some higher education	2.31	2.00–2.65	1.66	1.42–1.94
Higher education degree	3.66	2.91–4.59	2.03	1.59–2.60
Color/race				
Nonwhite	1.00	-	1.00	-
White	1.78	1.59–1.99	1.23	1.08–1.40
Region				
Southeast	1.00	-	1.00	-
North	0.29	0.24–0.34	0.35	0.29–0.42
Northeast	0.41	0.35–0.48	0.53	0.45–0.62
South	0.77	0.65–0.92	0.78	0.65–0.94
Midwest	0.60	0.51–0.70	0.62	0.53–0.73
Having health insurance				
No	1.00	-	1.00	-
Yes	3.57	3.07–4.16	2.60	2.20–3.07

* Adjusted for all variables in the table.

Of the women who received medical request for mammography, 5.4% (95%CI 4.8–6.0) were unable to perform the examination. This percentage was more than four times higher among those who had no private health insurance when compared to those who had (7.6%, 95%CI 6.7–8.6 versus 1.7%, 95%CI 1.2–2.3, respectively).

The most reported reasons for not being able to perform the exam after medical request were: not thinking it was necessary; having the test scheduled, but not yet performed; and not being able to schedule it (Table 4). The difference between those with private health insurance and SUS users who failed to schedule the mammography was great (3.7% and 20.0%, respectively). However, this observation should be viewed with caution because of the small number of women interviewed who composed some of the response categories for that question.

**Table 4:** Main reasons for not performing mammography after receiving medical request. Brazil, 2013.

Reasons	All			Without health insurance			With health insurance		
		
	n	%	95%CI	n	%	95%CI	n	%	95%CI
Scheduled examination	141	23.0	18.3–28.4	115	21.2	16.3–27.1	26	36.3	23.9–50.8
Did not think it was necessary	167	27.2	22.1–33.0	147	27.1	21.6–33.4	20	28.0	16.5–43.3
Failed to schedule	111	18.1	13.5–23.9	108	20.0	14.8–26.5	3	3.7	1.5–8.7

Among the interviewees, 74.8% reported receiving the test results less than a month after it was performed. This percentage was higher for those who have health insurance (86.6%). Although only 0.3% of the interviewees had not received the test results, this percentage decreases from 0.47% among SUS users to 0.05% among those with private health insurance (Table 5).

**Table 5:** Time interval between performing and receiving the result of mammography. Brazil, 2013.

Time interval	All		Without health insurance		With health insurance	
		
	%	95%CI	%	95%CI	%	95%CI
< 1 month	74.8	73.2–76.4	67.3	65.2–69.4	86.6	84.5–88.5
1**├** 3 months	19.0	17.6–20.5	25.1	23.3–27.1	9.4	7.8–11.3
3**├** 6 months	1.7	1.4–2.1	2.3	1.8–2.8	0.8	0.5–1.4
≥ 6 months	1.4	1.0–1.9	1.5	1.0–2.1	1.3	0.8–2.2
Not yet received	2.3	1.9–3.0	2.9	2.2–3.7	1.5	0.9–2.5
Never received	0.3	0.2–0.5	0.5	0.3–0.8	0.0	0.0–0.1

## DISCUSSION

The coverage self-reported by women from 50 to 69 years about the performance of mammography in the past two years in Brazil, from PNS 2013 data, was 60.0%. This proportion is higher than that verified by PNAD 2008 (54.2%)^9^, but still shows great variation between regions. Additionally, the coverage of mammography among women in the recommended age group (50–69 years) varies greatly between those who depend exclusively on SUS (51.0%) and those with private health insurance (79.5%).

In 2004, the Brazilian Ministry of Health released the first document of Consensus for Control of Breast Cancer, which established the age group of 50–69 years as target of the screening. This age group was confirmed as target, in 2015, in the Clinical Guidelines for Control of Breast Cancer, also approved by the Brazilian Ministry of Health, after extensive literature review and public consultation^15^. Although there is much discussion around this prioritization of age, current scientific evidence confirms that women in this age group are those that most benefit from the mammographic screening^5,16^.

This study allows evaluating, for the first time with national representation, fundamental steps and access barriers to ensure the effectiveness of a screening program, such as having the request made by a doctor and the reasons affecting this condition. Our results showed that 66.7% of the women interviewed had a medical request for mammography, but, again, we observed great variation between SUS users (59.4%) and those with private health insurance (83.9%). We draw attention, however, to the fact that the higher proportions of women with medical requests among those met by SUS belong to the age group of 50–59 and 60–69 years, suggesting that SUS services have a tendency to follow the recommendations of the Ministry of Health. Among women with private health insurance, the percentage of mammography request in the age group of 40–49 years (84.5%) is similar to that found among women of 60–69 years (83.5%), which are part of the target population of the screening.

The scientific evidence is clear in showing that the balance between the benefits and risks of mammographic screening is favorable for women between 50 and 69 years. Younger women tend to present a higher amount of false positive results, which may bring damages due to overdiagnosis and overtreatment^17^. The cumulative risk among women from 40 to 49 years, annually subjected to a mammography, of having a false positive result in 10 years is about 60%^18^.

The factors that were most positively associated with having a mammography medical request are: having private health insurance, high education level, and being white. Live in the North and Northeast regions reduced the chances of having a mammography medical request. Other studies conducted in the Country have already indicated the difficulty of access among women from the economically poorest regions of Brazil^19,20^. However, it is possible to observe that, once having the medical application, almost all women can perform the mammography. Only 5.4% of women who had medical request for mammography had not yet carried out the examination at the time of the interview. However, this percentage was more than four times higher among those who had no private health insurance when compared to those who had (7.6% versus 1.7%, respectively). Although this datum should be viewed with caution due to the small number of observations, the great difference found between SUS users and those with health insurance that failed to schedule the examination called our attention (20% and 3.7%, respectively).

Based on data recorded in the SUS information systems, it is possible to note the existence of major inequalities between the regions of the Country not only for mammography examination, but also for the essential steps to ensure the success of a screening program: access to diagnosis and treatment for women with suspicious screening tests^19^. As identified by the coverage self-reported in PNS, the data of the SUS health information systems confirm the North region, followed by the Northeast, as the regions that stand out by presenting lower possibility of early detection and access to surgery.

Although lower mammography coverage has been identified among women who depend on SUS, an expansion in the supply of mammography has been observed in the Country. Data from PNAD 2003 and 2008 had already shown an increase of 42% among women without health insurance, greater than that verified between those with health insurance^20^.

The data presented here strengthen what was already observed in other studies, which show that the access barriers for screening breast cancer are higher among the population living outside major urban centers and depending exclusively on services provided by SUS. Our results indicate that, perhaps, the first important access barrier to early detection of breast cancer is getting a medical request for performing mammography. Almost 70% of the interviewed 40-year-old or older women had a medical request for mammography, and this percentage was higher among those who had private health insurance. However, we observed that, once having the medical request, only 5% of women were unable to perform the examination; but, again, we noted inequality of access when the data were stratified by having private health insurance or not. Less than 2% of women covered by private health insurance failed to perform the examination after medical request.

It is important to note that the gains in relation to population mammographic screening should be considered and evaluated constantly in the light of scientific knowledge^21,22^, within what is proposed as organized strategy. Surely, there are plenty of existing inequalities in the access to health-care, and the screening depends on a number of conditions that begin with women’s awareness and professional compliance with the clinical guidelines currently in force. Therefore, the implementation of the organized screening for breast cancer can set the capacity for early detection of cases and thus direct efforts to the appropriate treatment for effectively decreasing mortality by breast cancer.

Efforts should be made to expand the actions of early detection of breast cancer, reducing the inequalities of access in all stages of this process and ensuring that all women can benefit from the appropriate treatment. Warning strategies to the problem and investment in the training of professionals in the health-care network, with rationalization of the provision of services, should be prioritized within the national cancer control policy and in the comprehensive care to women.

## Footnotes

a Ministério da Saúde (BR), DATASUS. Informações de Saúde. PNS – Pesquisa Nacional de Saúde – 2013: módulo de cobertura de mamografia entre mulheres de 50 anos ou mais. Brasília (DF); s.d. [cited 2017 Jan 20]. Available from: http://tabnet.datasus.gov.br/cgi/deftohtm.exe?pns/pnskb.def
